# Dilated Cardiomyopathy: Beware of Diet Drugs Slimming the Heart

**DOI:** 10.7759/cureus.36874

**Published:** 2023-03-29

**Authors:** Pradnya Brijmohan Bhattad, Pramukh Arun Kumar, Mahati Dasari, Akil A Sherif, Ajay K Mishra, Allen W Filiberti

**Affiliations:** 1 Cardiovascular Medicine, Saint Vincent Hospital, UMass Chan Medical School, Worcester, USA; 2 Internal Medicine, Saint Vincent Hospital, Worcester, USA; 3 Internal Medicine, Saint Vincent Hospital, Worcester , USA

**Keywords:** amphetamine related cardiomyopathy, nonischemic cardiomyopathy, phentermine/topiramate, heart failure with reduced ejection fraction, dilated cardiomyopathy

## Abstract

There have been rare reports of dilated cardiomyopathy from chronic use of phentermine/topiramate, although very limited data are available. Phentermine is an atypical amphetamine analog that has been contraindicated in patients with a history of cardiovascular disease. We present a case of nonischemic dilated cardiomyopathy in the setting of chronic phentermine/topiramate use, which is the most likely cause of her dilated cardiomyopathy.

## Introduction

Obesity, a well-known cardiovascular disease risk factor, predisposes individuals based on body fat mass and regional body fat distribution, negatively affecting cardiac structure and function. Given the increasing prevalence of obesity in the older population secondary to the increased lifespan and the younger generation, various management methods are available to aid in weight loss [1]. Pharmacotherapy is now a well-recognized option for weight loss, but paradoxically, it can have adverse effects related to the cardiovascular system. The manifestations could be diverse, ranging from arrhythmias, hypertension, and acute coronary syndromes to cardiomyopathy [2].

Dilated cardiomyopathy leads to the dilation of one or both ventricles with a reduced left ventricular ejection fraction below 40% caused by primary or secondary causes, with idiopathic being the most common [3]. Secondary causes such as infectious myocarditis, alcohol-induced, hypertensive, ischemic, and peripartum cardiomyopathy are well documented in the literature. Although drug-induced dilated cardiomyopathy has been reported with various classes of medications, the understanding of the mechanism is evolving and is found to be due to complex multifactorial mechanisms involving myocardial bioenergetics and has the potential to be reversed [4]. To the best of our knowledge, we describe the first known case of phentermine-topiramate extended-release causing dilated cardiomyopathy.

## Case presentation

A 68-year-old female with a past medical history of dyslipidemia and obesity presented with complaints of a three-week duration of progressively worsening dyspnea with minimal exertion, orthopnea, paroxysmal nocturnal dyspnea, and an unintentional weight gain of about 7 pounds. She denied any dizziness, diaphoresis, presyncope, syncope, chest pain, or palpitations. She did not report any prodromal symptoms of fevers, chills, cough, nausea, vomiting, or abdominal pain. A complete review of systems was unremarkable otherwise. She denied any recent sick contacts, travel, or immunizations. She reported the use of phentermine and topiramate, which were prescribed for sustained weight loss, and that she had been taking them for the preceding 1.5-2 years. She reported that she discontinued phentermine 37.5 mg/topiramate 25 mg once daily supplementation one week after the onset of her current presenting symptoms. Based on history, there was no evidence of familial cardiomyopathy. She denied any history of alcohol, substance, or tobacco use. Her blood pressure was 90/60 s mmHg, her heart rate was 90-110 s/minute, and her oxygen saturation was more than 95% on room air at the time of presentation. She was hospitalized for further management. A physical examination at the time of presentation revealed jugular venous distention elevated to the angle of the mandible, sinus tachycardia, a systolic murmur at the left sternal border, and diffusely diminished breath sounds over bilateral lung fields with bibasilar crackles.

**Figure 1:**
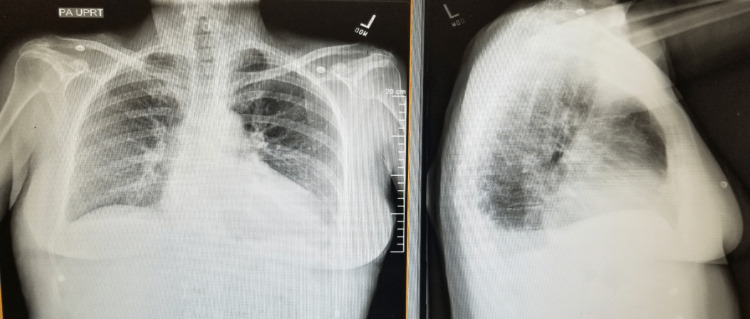
A chest X-ray at presentation demonstrating pulmonary vascular congestion.

A complete blood cell count, chemistry panel, electrolytes, serial cardiac biomarkers and enzymes, inflammatory markers such as erythrocyte sedimentation rate and C-reactive protein, and thyroid function studies were all within the normal reference range. Her NT-pro-brain natriuretic peptide level was elevated at 5000 pg/mL (ref < 125pg/mL). Her chest X-ray showed pulmonary vascular congestion (Figure 1).

**Figure 2:**
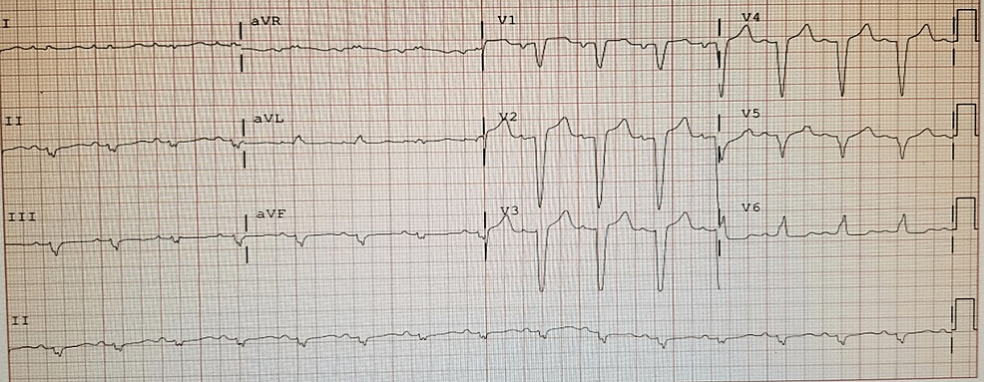
An ECG demonstrating sinus rhythm with a left bundle branch block and low voltage in the limb leads.

An electrocardiogram (ECG) showed sinus rhythm with a left bundle branch block and low voltage in the limb leads (Figure 2).

A transthoracic echocardiogram revealed severely reduced left ventricular systolic function with a left ventricular ejection fraction of 10-15%, severe global hypokinesis, a moderately dilated left ventricle, severe mitral regurgitation, severe tricuspid regurgitation, reduced right ventricular systolic function, and a dilated inferior vena cava. A viral screen, coronavirus disease-19 screen, urine toxicology screen, and blood and urine cultures were negative. A complete infectious work-up, including a coxsackie antibody panel, mycoplasma, human immunodeficiency virus, hepatitis C and hepatitis B screening, and serologies for adenovirus, cytomegalovirus, Epstein-Barr virus, and Lyme's disease, were negative.

She was aggressively diuresed during the hospitalization and did not require any inotropic or vasopressor support during this time. A coronary angiogram revealed normal coronary arteries during the hospitalization. Once she attained euvolemic status with aggressive diuresis and remained hemodynamically stable, she was discharged on a stable regimen with guideline-directed medical therapy with a beta-blocker and a renin-angiotensin system inhibitor.

She was recommended to permanently discontinue phentermine/topiramate. She was followed up as an outpatient with cardiac magnetic resonance imaging (MRI) three to four months later, which demonstrated a left ventricular ejection fraction of 30%, representing an improvement compared to prior imaging studies. There was no significant delayed gadolinium enhancement on cardiac MRI, with global severe hypokinesis of the left ventricle and at least moderate global hypokinesis of the right ventricle. The cardiac MRI demonstrated no evidence of previous myocardial injury or of any infiltrative, infectious, or inflammatory processes. Although her left ventricular ejection fraction improved from her baseline, given that it was severely reduced despite being on guideline-directed medical therapy, she underwent biventricular ICD implantation. She will be followed further as an outpatient.

## Discussion

Phentermine is an amphetamine derivative that stimulates the sympathetic pathway, which is used as an adjunct therapy for weight loss [5]. In 1959, phentermine was introduced as an anorectic in the United States. The primary mechanism of action of phentermine in treating obesity is hypothalamic stimulation to decrease appetite, which is facilitated by the non-selective stimulation of synaptic noradrenaline, dopamine, and serotonin release. The use of amphetamines has been restricted to only short-term therapy (up to 12 weeks) because of their potential for medication abuse and adverse side effects [6,7].

There have been documented cases of cardiovascular adverse events with phentermine, like primary pulmonary hypertension [8], valvulopathy [9-12], restrictive cardiomyopathy [13], cerebral hemorrhage [14], ischemic stroke [15], supraventricular tachycardia [6], stress-induced cardiomyopathy [16,17], ventricular fibrillation [18], and polymorphic ventricular tachycardia [19]. Topiramate has not been shown to be associated with increased cardiovascular risk but may be associated with a range of metabolic effects [6,9-11,17-19].

Although the exact mechanism by which phentermine causes cardiovascular adverse events is unknown, it is primarily thought to be due to its sympathomimetic properties. Other proposed mechanisms include serotonin acting as a vasoconstrictor and causing pulmonary vasoconstriction and coronary artery vasospasm secondary to increased adrenergic activity. There is also evidence of chronic exposure to sympathomimetic drugs resulting in myocardial injury [20-22].

FDA approved phentermine-topiramate extended-release in 2013 for the chronic treatment of obesity in selected patients. There are increasing cardiovascular concerns in patients receiving a combination of phentermine-topiramate extended-release, signifying the real-world safety of this contemporary anti-obesity medication [2,23].

## Conclusions

Management of obesity using pharmacotherapy is increasing in utility, and the side effect profile of the anti-obesity medications is yet to be thoroughly studied. Our patient had dilated cardiomyopathy associated with the use of phentermine for obesity. This case emphasizes the need for heightened post-marketing surveillance of the phentermine-topiramate drug combination for its cardiovascular side effect profile.
